# Deep brain stimulation in children with acquired dystonia

**DOI:** 10.3389/fneur.2026.1735832

**Published:** 2026-02-25

**Authors:** Lea Hagelschuer, Anne Koy

**Affiliations:** 1 Department of Pediatrics, Faculty of Medicine and University Hospital Cologne, University of Cologne, Cologne, Germany; 2 Center for Rare Diseases, Faculty of Medicine and University Hospital Cologne, University of Cologne, Cologne, Germany

**Keywords:** acquired dystonia, childhood, DBS, deep brain stimulation, dyskinetic cerebral palsy

## Abstract

The aim of this review is to present the current state of knowledge on deep brain stimulation (DBS) in pediatric patients with acquired dystonia. We summarize the short- and long-term effects of DBS on motor and non-motor domains and discuss potential factors influencing treatment response and patient selection. Furthermore, in view of the limitations of the existing data future perspectives are discussed, which could contribute to an improved understanding of disease mechanisms and outcome predictors in order to optimize the treatment by invasive neuromodulation in these often complex disabled patients.

## 1 Introduction

### 1.1 Acquired dystonia

Dystonia is defined as *a movement disorder characterized by sustained or intermittent abnormal movements, postures, or both. Dystonic movements and postures are typically patterned and repetitive and may be tremulous or jerky. They are often initiated or worsened by voluntary action, and frequently associated with overflow movements.* The etiology can be genetic, acquired, or unknown (1).

The most common cause of acquired dystonia in childhood is dyskinetic cerebral palsy (DCP) (2, 3). Therefore, the focus of this review will be on this disease entity.

According to the recently proposed updated definition by Dan et al. (4), cerebral palsy (CP) is an *early-onset lifelong neurodevelopmental condition characterized by limitations in activity due to impaired development of movement and posture, manifesting as spasticity, dystonia, choreoathetosis, and/or ataxia*. The overall prevalence of CP in high-income countries is 1.5–3.1 per 1,000 live births. DCP accounts for up to 15% of CP cases, making it the second most common type after spastic CP (5, 6).

DCP most often arises from hypoxic–ischemic encephalopathy (HIE) due to perinatal asphyxia in near to term neonates, additional causes include kernicterus, intracranial hemorrhage, perinatal stroke, and central nervous system infections during the neonatal period (3, 7). Postneonatal causes such as cardiorespiratory arrest or near drowning are rare (3). Reflecting the diverse etiologies, DCP shows marked phenotypic heterogeneity (8). Distinct lesion patterns and localizations within the developing brain result in variable symptoms (9). Comorbidities in DCP are frequent and include motor and non-motor symptoms such as intellectual disability (>50%), orthopedic deformities (58% of children with dystonia) (10), spasticity (34.3% in children with acquired dystonia) (9), pain (38.1% of children with dystonia) (11, 12), anarthria or dysarthria, epilepsy, visual impairment, hearing loss, impaired sleep, constipation and others (3, 13). Dystonic symptoms in DCP frequently fluctuate in severity and can be exacerbated by several factors such as emotional distress, infections, pain, sleep deprivation, or constipation (3).

While DCP represents the predominant form of acquired dystonia in childhood, other etiologies must also be considered, such as drug- or toxic-induced dystonia, structural lesions due to vascular events, trauma, tumor, inflammatory or infectious diseases of the central nervous system, neurometabolic disorders, or hypoxic events later in life (14). In a pediatric cohort of 379 patients with acquired brain injury (median age 4.8 years), 12 patients developed dystonia (median age 9.1 years). In most cases dystonia occurred after traumatic brain injury (58.3%), due to hypoxia/anoxia (16.7%), hemorrhage, immune/inflammatory causes, and stroke (each 8.3%) (8). In adults, the main causes for acquired dystonia include tardive dystonia, vascular lesions after stroke, head trauma, multiple sclerosis, or autoimmune diseases (15). Data on DBS in these non-DCP acquired forms, particularly in the pediatric population, are very limited. Robust quantification of etiology-specific response rates is lacking, as available data are restricted to single case reports or case series (16), or are usually pooled under the category ‘acquired’ in meta-analyses (17).

Compared to genetic forms, CP generally shows a non-progressive, yet persistent, but often disabling course. However, while in many patients with DCP the motor pattern remains relatively stable for years, the functional abilities may further deteriorate with increasing age due to secondary musculoskeletal complications, such as contractures or dislocations of joints (3, 9). In contrast, dystonia due to acquired injury of the mature brain later in life, e.g., after stroke or trauma, may initially fluctuate or worsen over months before reaching a stable, chronic state. Spontaneous improvement of symptoms is rare but occurs occasionally in early post-acute phases (8). Overall, comprehensive long-term studies on the disease courses of patients with DCP are rare, which makes the interpretation of treatment effects difficult, since even the preservation of functions can already be a therapeutic success. Patients with acquired dystonia are, on average, more severely affected by motor impairment than those with genetic dystonia: in a European cohort of patients with dystonia, 87% of patients with acquired dystonia were classified as GMFCS level IV/V, compared to 25% in genetic dystonia (9). In general, the level of motor function in patients with acquired dystonia is often low, especially when other neurological symptoms such as spasticity, ataxia, or central hypotonia are also present (9, 10).

Status dystonicus (SD) is a movement disorder emergency with “*increasingly frequent and severe episodes of generalized dystonia, which necessitate urgent hospital admission”* (18). Approximately 35% of SD cases occur in patients with acquired dystonia (19), with reported mortality rates ranging from 10 to 12.5% (20).

### 1.2 Pathophysiology

The pathophysiology of dystonia is not yet fully understood. Most of our understanding derives from studies of isolated or genetic forms, whereas the mechanisms underlying acquired dystonia are comparatively even less well understood. Dystonia is considered as a network disorder involving the basal ganglia-thalamo-cortical network with input of the cerebellum, cortical and subcortical areas and the brainstem (21, 22). It is associated with impaired sensorimotor integration, reduced inhibitory control, and abnormal plasticity, whereas it remains unclear to what extent these changes are primary or compensatory (23). Resting-state functional MRI (rs-fMRI) and diffusion-based connectomic studies confirm the concept of dystonia being a network disorder, demonstrated by dysfunction of several brain areas such as the basal ganglia, thalamus, cerebellum, brainstem, and cortex (24–26).

Genetic dystonia is caused by impaired gene function affecting processes such as dopamine / neurotransmitter signaling, intracellular trafficking, abnormalities of ion homeostasis, intracellular stress, energy metabolism, degradation, and others (27). Although the genetic landscape of dystonia is broad, converging evidence indicates that several of these diverse defects share common pathways within the striato-thalamo-cortical and cerebello-thalamo-cortical networks (21).

In acquired dystonia, however, neuronal networks are impaired by structural lesions, which may be multifocal and can involve several functionally connected brain regions (26). Typical MRI patterns can be classified according to the MRI Classification System (MRICS), which links imaging findings to specific time windows of brain injury. In patients with DCP, gray matter injuries predominate, according to lesions occurring during the third trimester or perinatally, classified as MRICS-C (28). Lesions are typically found in the basal ganglia [especially globus pallidus, nucleus subthalamicus (STN), putamen] and thalamus (50%) (29), and to a lesser extent cortico-subcortical (20%) or in the white matter (28%) (26). Beyond DCP other etiologies can have different pathophysiological mechanisms and result in various lesion patterns, e.g., inflammatory processes may affect basal ganglia, thalamic, or cerebellar function through immune-mediated mechanisms, including cytokine-mediated neuroinflammation, microglial activation, and autoantibodies. MRIs can show corresponding T2/FLAIR hyperintensities in these regions (30). Dystonia due to traumatic brain injury arises predominantly from contralateral lesions of the basal ganglia due to rotational shear injury, hemorrhages, or ischemia of lenticulostriate arteries, causing gliosis, cavitation, and with characteristic T2-hypointense hemosiderin deposits in globus pallidus internus (GPi), putamen and occasional thalamus (31). Toxic or metabolic insults can provoke mitochondrial dysfunction and bilateral symmetric lesions the basal ganglia (32). Vascular events of the basal ganglia and/or the thalamus disrupt pallido-thalamo-cortical networks via diaschisis, manifesting as acute DWI hyperintensities and later on chronic gliosis/lacunar defects or SWI-positive hemosiderin (33).

An analysis of case series of lesion-induced dystonia (e.g., due to stroke, hemorrhage, or tumor) showed a link between lesion location and the pattern of dystonic symptoms. Limb dystonia is most often associated with basal ganglia lesions, hand dystonia with thalamic lesions, and cervical dystonia with lesions in the brainstem or cerebellum (34). These findings support the concept that distinct dystonia phenotypes reflect impairment of specific functional circuits within the motor network (33, 35).

Microstructural network alterations have been demonstrated in patients with dystonia of various etiologies (36). A recent study on connectivity patterns in patients with dystonia demonstrated higher fiber density between GPi and putamen in patients with acquired dystonia compared to patients with genetic forms of dystonia, suggesting enhanced striato-pallidal connectivity, which could indicate an overactivation of the direct pathway. This enhanced connectivity may impair inhibitory control within the motor circuits, contributing to the hyperkinetic and hypertonic movements in DCP (36). Complementing these structural findings, literature-based lesion-derived network map analyses on patients with DCP has shown, that causative lesions are functionally connected to a wide network including the brainstem, cerebellum, basal ganglia, cingulate and sensorimotor cortices. The strongest connectivity was found for the motor thalamus [including the mediodorsal nucleus and ventro-intermediate (Vim)/ventro-oral-posterior nuclei (Vop)], highlighting the central role of this particular hub within the motor network (26).

### 1.3 Pharmacotherapy

The treatment of acquired dystonia is challenging and requires an interdisciplinary approach. Supportive therapies are central to treatment and include physiotherapy, occupational therapy, speech therapy, orthoses and other assistive devices. The primary goals should be patient-centered and include improvement of mobility and functional abilities and enhancing long-term quality of life (37).

The response to pharmacotherapy in patients with acquired dystonia is heterogeneous and overall poorer than in genetic forms of dystonia. Side effects are often hardly tolerable. Evidence for pharmacotherapy is limited so far, with most medications showing little to no effect on motor function or dystonia severity (38, 39). A recently published clinical practice guideline by the AACPDM (American Academy for Cerebral Palsy and Developmental Medicine) recommends the use of oral / enteral baclofen as first-line treatment, and as second line gabapentin and clonidine, especially for pain reduction and improved sleep in DCP patients with generalized dystonia. Whereas in patients not responsive to the oral medication intrathecal baclofen or DBS should be considered to improve dystonia, goal attainment, pain, and quality of life, while in focal or segmental dystonia the use of BoNT-A is suggested (38, 40–42). However, these recommendations are based on a limited level of evidence (40, 42). In clinical practice, baclofen, trihexyphenidyl, gabapentin, and benzodiazepines are commonly used in order to manage tone and pain (43). Despite oral baclofen being the preferred first-line agent, no controlled studies confirm its efficacy for dystonia in CP, and there is little consensus on subsequent therapies (38).

## 2 Deep brain stimulation in acquired dystonia

Deep brain stimulation (DBS) is an invasive neuromodulation method, by which depth electrodes are stereotactically implanted into specific deep brain nuclei and connected to a subcutaneous pulse generator (44). DBS delivers high-frequency electrical pulses to the target region, modulating neuronal activity locally as well as along connected distributed brain networks achieving a reversible and titratable therapeutic effect (45, 46). Initially introduced for the treatment of refractory Parkinson’s disease in the late 1980s, DBS has since been applied for several movement disorders, including dystonia and tremor, as well as selected neuropsychiatric conditions and treatment-refractory epilepsies (47–49). The most common stimulation targets for dystonia are the GPi, the STN, and the thalamus, chosen according to the underlying disorder and symptom profile (50). Over the last three decades DBS also has been increasingly applied in pediatric patients with pharmacorefractory genetic and acquired dystonia.

In most cases, DBS is performed as an elective procedure, except for status dystonicus (SD). In this life-threatening condition, DBS can also be considered as an emergency intervention. The number of reported cases is limited, but in most of the patients DBS had a beneficial effect on the disease course (51). The effects occurred within days or weeks, alleviating the weaning from sedative and anaesthetic agents, and in some cases dystonia severity improved compared to baseline. Some authors therefore, propagate the rapid and early application of DBS in SD in order to reduce the risk of mortality and long-term morbidity associated with this condition (52). However, DBS surgery in a patient with SD poses an increased risk of hardware defects and other serious complications, therefore the indication needs to be weighed up carefully, especially in children (53). An alternative to DBS in SD can be lesioning surgery. Pallidotomy and thalamotomy have largely been replaced by DBS over the last decades, but can be considered in SD if DBS is not available (54).

### 2.1 Assessment of treatment effects

Previous studies have used various clinical scores for the standardized assessment of the effectiveness of DBS in acquired dystonia (55). The Burke-Fahn-Marsden Dystonia Rating Scale (BFMDRS) is the most commonly applied assessment. It was originally developed for adult patients with isolated genetic dystonia (56). The Barry-Albright Dystonia Scale (BADS) is validated for pediatric patients with CP and has also been commonly applied (57).

The Dyskinesia Impairment Scale (DIS) has recently been conceived and enables the assessment of the complex hyperkinetic and hypertonic components of DCP as it evaluates dystonia and choreoathetosis in action and rest (58).

All the mentioned scales focus solely on motor impairments, and may not be able to reliably capture small yet potentially meaningful changes. To address this limitation, additional assessments have been increasingly implemented in order to detect subtle changes in broader motor and non-motor domains, which can be relevant to the individual patient. These include, in particular, the Canadian Occupational Performance Measure (COPM) (59–61), the Gross Motor Function Measure-66 (GMFM-66) (62), and quality-of-life scales like the CPCHILD (63). Other relevant domains include parental burden (64), psychosocial wellbeing (65), pain intensity (66), speech, swallowing, and communication abilities (67–69). Changes in other domains such as cognitive development, sleep quality, body mass index (BMI), anti-dystonic medications and other supportive treatments can also be informative as indirect indicators of DBS effectiveness (70).

### 2.2 Effects on motor domains

DBS has been proven safe and effective in certain forms of genetic dystonia, e.g., DYT-TOR1A, -KMT2B, -GNAL and GNAO1 (71–75). According to a recent meta-analysis a mean reduction of the BFMDRS Motor Score (BFMDRS-M) in genetic and idiopathic dystonia was 57.2% (70). Effects in patients with acquired dystonia, such as DCP, are significantly more variable and less distinct (2, 76–78) (Table 1). In acquired dystonia, the mean improvement of the BFMDRS-M reported across most studies is approximately 20–24% (2, 16, 55, 59, 61, 77, 79–86).

**Table 1:** Overview of published data (2009–2022) on DBS effects on dystonia.

Reference	Study type	Etiology of acquired dystonia	DBS target	n=	Age at implantation (years)	Follow-up	Outcome measure	Improvement
Koy et al., 2022 (88)	Prospective multicenter study	DCP due to HIE	GPi	16	13.4 (mean)	36 months	DIS/BFMDRS-M/BFMDRS-D	14.6%/no significant change in BFMDRS
Vidailhet et al., 2009 (82)	Prospective multicenter study	DCP due to HIE	GPi	13	20–44	12 months	BFMDRS-M	24.4%
Koy et al., 2017 (80)	Multicenter registry study	Mixed etiologies^a^	GPi, STN, Vim	16	10.4 (mean)	24 months (mean)	BFMDRS-M	10.7%
Mandarano et al., 2022 (93)	Cohort study	Mixed etiologies^b^	GPi	9	7–43	12 months	BFMDRS-M/BFMDRS-D	19.9%/7.8%
Lumsden et al., 2013 (87)	Cohort study	Mixed etiologies^c^	GPi	24	3–20	12 months	BFMDRS-M	7.3%
Romito et al., 2015 (79)	Case series	DCP due to HIE	GPi	15	15–47	4.4 years (mean)	BFMDRS-M/BFMDRS-D	49.5%/30%
Gimeno et al., 2014 (61)	Case series	Mixed etiologies^d^	NR	14	3–18	12 months	BFMDRS-M	4%
Koy et al., 2014 (81)	Case series	Mixed etiologies^e^	GPi	8	16–33	44.5 months (mean)	BFMDRS-M	no significant change
Keen et al., 2014 (83)	Case series	Mixed etiologies^f^	GPi	5	8–17	26.6 months (mean)	BFMDRS-M	28.5%
Marks et al., 2013 (84)	Case series	Mixed etiologies^g^	GPi	9	7–15	12 months	BFMDRS-M	24.3%
Marks et al., n (85)	Case series	DCP due to mixed etiologies	GPi	15	7–26	6 months	BFMDRS-M/BFMDRS-D	25.46%/8.95% (<16 years 37.84% /14.44%)
Elia et al., 2018 (2)	Systematic review	DCP due to mixed etiologies	GPi, STN, Vim	124	3–47	2 months–11 years	BFMDRS-M	1.2–49.5%, mean 20%
Bohn et al., 2021 (38)	Meta-analysis	DCP due to mixed etiologies	NR	168	Children and adults	6 months–4.5 years	BFMDRS-M	16.8%
Elkaim et al., 2019 (17)	Meta-analysis	DCP due to mixed etiologies	GPi, STN, thalamus, internal capsule, pedunculo-pontine nucleus	59	14.0 (median)	12 months (median)	BFMDRS-M/BFMDRS-D	11.1%/3.5%
		DCP due to Kernicterus		8	12.2 (median)	12 months (median)	BFMDRS-M/BFMDRS-D	10.5%/3.5%
		Post-stroke		3	10.3 (median)	12 months	BFMDRS-M	11.2%
		Post-infectious		2	14.5 (median)	9 months	BFMDRS-M	25.8%
		Nigrostriatal necrosis		2	5 (median)	12 months	BFMDRS-M/BFMDRS-D	26.6%/no change
		Post-traumatic		1	21	12 months	BFMDRS-M/BFMDRS-D	72%/60%
		Metabolic		1	13	6 months	BFMDRS-M/BFMDRS-D	3.6%/3.8%
Koy et al., 2013 (77)	Meta-analysis	DCP due to mixed etiologies	GPi, STN, Vim, Voa	68	5–46	12 months (median)	BFMDRS-M/BFMDRS-D	23.6%/9.2%

BFMDRS-M, Burke-Fahn-Marsden Dystonia Rating Scale, motor score; BFMDRS-D, Burke-Fahn-Marsden Dystonia Rating Scale, disability score; DP, dyskinetic cerebral palsy; DIS, Dyskinesia Impairment Scale; GPi, globus pallidus internus; HIE, hypoxic–ischemic encephalopathy; NR, not reported; STN, nucleus subthalamicus; Vim, nucleus ventralis intermedius; Voa, nucleus ventralis oralis anterior.

a perinatal asphyxia *n* = 9, trauma *n* = 2, hypoxic brain injury *n* = 1, infection *n* = 2, hemorrhage *n* = 1, kernicterus *n* = 1.

b HIE *n* = 6, kernicterus *n* = 1, pantothenate kinase-associated neurodegeneration (PKAN) *n* = 1, Lesch–Nyhan syndrome *n* = 1.

c ex-preterm *n* = 9, cerebral palsy *n* = 6, kernicterus *n* = 4, glutaric aciduria *n* = 3, nigrostriatal necrosis *n* = 2.

d ex-preterm *n* = 7, kernicterus *n* = 4, HIE *n* = 2, periventricular leukomalacia (PVL) *n* = 1.

e HIE *n* = 7, kernicterus *n* = 1.

f HIE *n* = 2, hemorrhage *n* = 1, infection *n* = 1, kernicterus *n* = 1.

g HIE *n* = 3, ex-preterm *n* = 3, kernicterus *n* = 1, cerebral dysgenesis *n* = 1, unknown *n* = 1.

When stratifying according to etiology, reported outcomes in acquired dystonia vary considerably as summarized in the meta-analysis by Elkaim et al. (17), summarizing data from several primary reports (16, 78, 80, 87). In children with acquired dystonia due to kernicterus (*n* = 8), DBS resulted in a median improvement of the BFMDRS-M of 10.5% with minimal change in the BFMDRS disability score (BFMDRS-D) of 3.5% at a median follow-up of 12 months. In a small cohort (*n* = 3) of stroke-associated dystonia a median improvement of 11.2% in the BFMDRS-M was reported after a follow-up of 12 months. Two patients with dystonia after infections of the central nervous system showed a median improvement of 25.8% of the BFMDRS-M after a follow-up of 9 months. In two patients with nigrostriatal necrosis, a median BFMDRS-M improvement of 26.6% without any improvement of the BFMDRS-D was reported after 12 months (87). One patient with post-traumatic dystonia achieved marked motor (72%) and disability (60%) improvements after 12 months of follow-up. One patient with metabolic dystonia showed only minimal change in the BFMDRS-M of 3.6% and in the BFMDRS-D of 3.8% after 6 months.

Overall, the evidence for DBS in acquired dystonia, including DCP, remains limited, as it is predominantly based on retrospective case series or smaller cohort studies, often without standardized assessment of clinical response. Notably, two prospective studies have provided more robust data. In a multicenter prospective pilot study of 13 adults with dystonia-choreoathetosis due to CP, a mean BFMDRS-M improvement of 24% after 1 year of GPi-DBS could be demonstrated, accompanied by significant gains in function, pain, and quality of life, with stable cognition and mood (82). Another prospective, multicenter trial investigated 16 pediatric DCP-patients undergoing GPi-DBS and showed significant improvement of the DIS after 36 months on long-term follow-up. There was a considerable interindividual variability in treatment response, without any significant changes in the BFMDRS-M and quality of life scales (88).

These observations are corroborated by several systematic reviews and meta-analyses, which include pediatric and adult patients, particularly with DCP: A systematic review by Elia et al. (2) identified 12 mostly retrospective studies on DBS in patients with DCP (124 patients, age 3.5–47 years, follow-up 2 months–11 years). Most patients underwent bilateral GPi-DBS, most studies used the BFMDRS-M as the primary outcome, reporting highly variable improvements ranging from 1.2 to 49.5% with a mean improvement of 20% (2). Elkaim et al. (17) performed a meta-analysis from 72 studies (59 patients with DCP ≤ 21 years) with overall poor response in patients with acquired dystonia (median BFMDRS-M change 11.1% at 12 months). Only 27% of DCP patients achieved clinically significant (<20%) improvement (17). Bohn et al. (38) analyzed 19 studies (168 patients with DCP, follow-up 6 months–4.5 years) providing low certainty evidence of DBS reducing dystonia (BFMDRS-M −16.8%) with potential improvements in pain/comfort, individualized goal attainment, and quality of life.

Long-term follow-up data is only available for adult patients with isolated generalized or segmental dystonia, showing stable effects after 10-year postoperative follow-up in 31 patients (89). A single-case study demonstrated that beneficial effects of DBS persisted even after discontinuation of stimulation due to battery depletion after 6.5 years. This observation supports the concept of dystonia as a network disorder and suggests that DBS may induce neuroplastic changes with a potential disease-modifying impact (90). Reports on follow-up beyond 10 years postoperative are not available for pediatric patients with acquired dystonia.

### 2.3 Effects on non-motor domains

There is only very limited data on the effects of DBS on neuropsychological aspects in patients with acquired dystonia. According to the available data on adult patients, GPi-DBS does not seem to impact mood or cognition (79, 82), whereas there was a positive impact on depression as well as on paranoid and psychotic symptoms reported (82). Beneficial effects could be observed on health-related quality of life (e.g., SF-36), particularly in the physical domains, while mental and emotional aspects are less consistently affected (79, 81, 91). In a pediatric cohort, quality of life assessed by the CPCHILD did not change significantly after 12 months, though treatment outcomes varied substantially between individuals (88). Speech and swallowing appear largely unaffected by DBS in acquired dystonia, with no consistent evidence for clinically relevant improvement (81, 92) (Table 2).

**Table 2:** Overview of published data (2009–2025) on non-motor domain outcomes in patients with acquired dystonia.

Reference	Study type	Etiology of acquired dystonia	DBS target	n=	Age at implantation (years)	Follow-up	Outcome measure	Effect
Koy et al., 2022 (88)	Prospective multicenter study	DCP due to HIE	GPi	16	13.4 (mean)	12 months	CPCHILD, COPM, SF-36	CPCHILD: 10.7% improvement, not significant COPM performance: 64.0% improvement SF-36 physical health: 17.1% improvement, not significant SF-36 mental health: 3.1% improvement, not significant
Vidailhet et al., 2009 (82)	Prospective multicenter study	DCP due to HIE	GPi	13	20–44	12 months	SF-36, SCL-90, Beck depression inventory, cognitive function	Reduced pain, improved mental health, no cognitive impairment, no effect on mood; no further need for movement disorder medication; one patient no longer requiring antidepressant medication
Bernardi et al., 2025 (92)	Pro- and retrospective multicenter study	DCP due to mixed etiologies^a^	GPi	14	7–18	12 months	Frenchay Dysarthria Assessment	No significant changes in speech and swallowing
Mandarano et al., 2022 (93)	Cohort study	Mixed etiologies^b^	GPi	9	7–43	12 months	CHQ-PF50, cognitive function	CHQ-PF50, psychosocial: 28.6% improvement Cognition improved or stable, semantic fluency improved or stable
Perides et al., 2020 (12)	Cohort study	Mixed etiologies^c^	GPi, STN	37	3–19	12 months	CPCHILD, NPRS, PPP	CPCHILD: 18% improvement; Significant reduction in pain frequency and severity, reduced analgesic use, increased proportion without pain
Owen et al., 2017 (94)	Cohort study	Mixed etiologies^d^	GPi	40	12.5 (mean)	1–3 years	cognitive function	Cognition remained stable, improvement in picture completion subtest
Romito et al., 2015 (79)	Case series	DCP due to HIE	GPi	15	15–47	4.4 years (mean)	SF-36, cognitive function	SF-36: 81% improvement No cognitive impairment, or psychiatric disorders
Koy et al., 2014 (81)	Case series	Mixed etiologies^e^	GPi	8	16–33	44.5 months (mean)	Frenchay Dysarthria Assessment	Speech and swallowing mostly unchanged, subjective symptom improvements
Aihemaitiniyazi et al., 2023 (91)	Meta-analysis	Mixed etiologies	GPi, STN Vop, Vim	156	34.3 years (mean)	2.2 years (mean)	SF-36	Significant improvements in physical (pronounced) and mental health

CHQ-PF50, Child Health Questionnaire parent form; COPM, Canadian Occupational Performance Measure; CPCHILD, Caregiver Priorities and Child Health Index of Life with Disabilities; DCP, dyskinetic cerebral palsy; GPi, globus pallidus internus; HIE, hypoxic–ischemic encephalopathy; NPRS, Numeric Pain Rating Scale; PPP, Pediatric Pain Profile; SCL-90, Hopkins Symptom Checklist; SF-36, short-form 36 health survey; STN, nucleus subthalamicus; Vim, nucleus ventralis; Vop, nucleus ventralis oralis posterior.

a HIE *n* = 13, infection *n* = 1.

b HIE *n* = 6, kernicterus *n* = 1, pantothenate kinase-associated neurodegeneration (PKAN) *n* = 1, Lesch–Nyhan syndrome *n* = 1.

c including DCP *n* = 24.

d DCP *n* = 22, NBIA *n* = 5, glutaric aciduria type 1 *n* = 3, Lesch–Nyhan syndrome *n* = 2, mitochondrial disorder *n* = 2, cerebrovascular accident *n* = 1, congenital neuromuscular disorder *n* = 1, HIE *n* = 1, infection *n* = 1, traumatic brain injury *n* = 1, brain malformation *n* = 1.

e HIE *n* = 7, kernicterus *n* = 1.

Perides et al. (2020) systematically assessed dystonic pain before and 1 year after DBS in children and young people, including 24 cases (38%) with DCP. Across the cohort, pain frequency and severity improved significantly: the proportion experiencing persistent or very severe pain decreased by 24.5% according to patients, while parents reported a decrease by 30%. The proportion of patients experiencing no pain increased by 28.6% reported by patients and 24% reported by parents. The number of daily used analgesic medications also declined from 34/63 to 9/63 after 12 months of treatment. The number of patients not requiring any more analgesia increased from 13/63 to 42/63 (12).

There are only two retrospective trials investigating effects on cognitive function in children with acquired dystonia. Cognition generally appears to remain stable after DBS, with no indication of global decline and occasional improvements in specific domains. Reported gains were mainly observed in perceptual reasoning, whereas other domains, such as overall intellectual ability and memory, largely remained unchanged (93–95).

### 2.4 Target selection

For genetic forms of dystonia, implantation of electrodes in the GPi has been the most widely used approach for which the most comprehensive and long-term clinical data are available (49, 70). GPi-DBS is also been associated with little to missing side effects on mood and cognition (82, 89, 96). Data on alternative targeting in dystonia remains limited.

Selecting the appropriate target for patients with acquired dystonia is more challenging due to multifocal structural brain changes and heterogeneous clinical presentations. So far, in acquired dystonia, GPi is the most studied target site, too, whereas other targets have only been investigated in single case reports or smaller case series (22, 97). Reasons that may prompt consideration of alternative DBS targets include difficulty in electrode placement due to structural lesions or insufficient symptom improvement following GPi stimulation. In such cases, the STN may represent an alternative target (98). Two studies comparing GPi- and STN-DBS in isolated dystonia confirmed that both targets are safe and effective. STN-DBS lead to faster clinical effects, with improvements of the BFMDRS-M of 63–65% (*n* = 12–16) versus 38–48% for GPi (*n* = 14–17) after 6-months follow-up, and was superior for ocular, generalized, and upper-limb dystonia, whereas GPi-DBS was more effective for tonic and axial or trunk symptoms (e.g., trunk improvement 94% vs. 82%). Both targets improved disability, quality of life, and mood, and neither had a significant impact on cognition (99–101). A meta-analysis of 10 studies of STN DBS in acquired or neurodegenerative dystonia in 146 adult patients reported a mean BFMDRS-M improvement of 66% (range 28–98%) at an average follow-up of 20 months (102). However, the STN is anatomically smaller and functionally more complex: the dorsolateral, predominantly motor part borders directly on the ventral portion with limbic and associative functions. Precise imaging with diffusion tensor imaging (DTI) and target planning are therefore essential to avoid unwanted cognitive or affective side effects, especially in patients with pre-existing neuropsychological abnormalities (103). Other alternative targets include thalamic regions such as the Voa/Vop or Vim, which have been implanted in adult patients with dystonic tremor, writers’ cramp or acquired dystonia (104, 105). In a phase I trial, four patients (9–19 years) with severe acquired dystonia received bilateral Vop/Vim thalamic DBS and were followed for 12 months. The procedure was well tolerated, with modest motor improvement (BFMDRS-M 21.5%, BADS 1.6%) and sustained gains in disability and quality of life (97).

In a case series on three patients with genetic dystonia, stimulation of the pedunculopontine nucleus (PPN) also resulted in a clinically relevant improvement in motor symptoms and quality of life (106).

In a pilot study, three young patients (14–22 years) with DCP due to hypoxic injury to the basal ganglia underwent bilateral cerebellar DBS targeting the dentate nucleus and the cerebellar outflow pathway. The procedure was well tolerated and led to variable but meaningful motor improvements (BFMDRS-M 19–40%) alongside subjective benefits in coordination, gait, speech, and muscle tone (22).

Although evidence for these alternative targets is limited to date, current findings suggest potential for individualized, network-based target selection, particularly in patients with pronounced structural lesions or treatment-refractory disease. Animal experimental data further shows that pallidal stimulation can normalize abnormal cerebellar activity via network connections (107).

In order to identify prognostic markers to optimize targeting and stimulation parameters, functional analysis of the motor network becomes increasingly relevant. In cervical dystonia, structural connectivity to the motor putamen and optimal electrode position within the pallido-thalamo-cortical networks correlates with clinical outcomes (108). Complementarily, in an adult cohort with segmental and generalized dystonia (not acquired), cortical integrity of the sensorimotor and visuomotor areas (as measured by cortical thickness) correlates with clinical outcome after DBS (109).

The variability of optimal stimulation sites may at least partly be explained by network-specific differences within the target area. Previous studies have investigated several optimal stimulation sites within the pallidal region in adult patients with isolated dystonia (110, 111). According to a large study by Reich et al. investigating 105 adult patients with cervical and generalized dystonia undergoing DBS by probabilistic mapping of the best stimulation effects, the ventroposterior GPi and adjacent subpallidal white matter were identified as the regions for optimal outcome (112). Assuming that the stimulation sites seem to follow somatotopic regions within the pallidum, Horn et al. investigated 80 adult patients with dystonia, who underwent GPi-DBS by sweetspot and network mapping. They could demonstrate that optimal stimulation sites map to different areas within the target, adjacent fibre tracts, and whole-brain networks, depending on cervical vs. generalized dystonia (111). Voxels in the ventral motor area of the GPi were associated with the best improvements of the cervical cohort, whereas voxels at a more anterior and dorsal subregion of the GPi were associated with best improvements in the cohort with generalized dystonia. Furthermore, Horn et al. suggest that specific connections play a key role for treatment outcome. While modulation of the pallido-thalamic tracts leads to optimal improvement in generalized dystonia, stimulation of the striato-pallido-fugal axis leads to improvement in cervical dystonia. Functional network analysis to different regions in the context of best improvement, revealed a positive connectivity to the cerebellum and a negative connectivity to the somatomotor cortex in patients irrespective of the type of dystonia (111). Overall, there are marked differences in optimal stimulation sites embedded in a somatotopic structure of the pallidum and the adjacent fibre bundles, therefore treatment success seems to not only depend on electrode position, but also on the precise volume of stimulation within the target zone (111, 112). Yet, such data on DBS effects on network connectivity in patients with acquired dystonia are missing.

### 2.5 Implantation and stimulation

**Figure 1:**
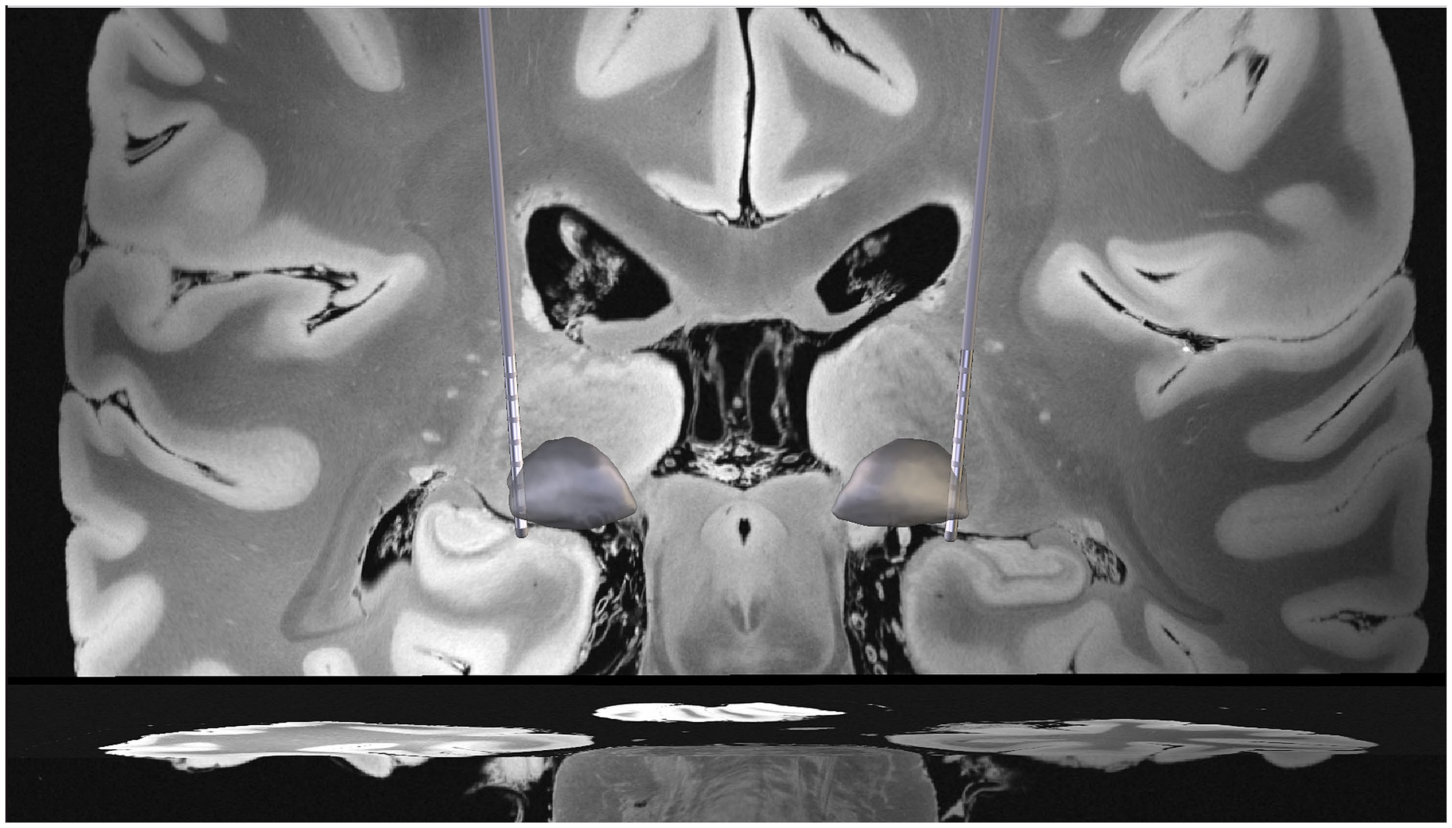
Coronal MRI view illustrating bilateral reconstruction of globus pallidus internus (GPi) deep brain stimulation electrodes in a pediatric patient with dystonic cerebral palsy, showing electrode trajectories and contact positions relative to pallidal anatomy.

DBS implantation is performed using a stereotactic frame or frameless navigation for target localization, followed by electrode placement and connection to a subcutaneous implantable pulse generator (IPG) (113, 114). Intraoperative microelectrode recordings of discharge patterns and testing of stimulation to assess stimulation thresholds is commonly performed, but may be susceptible to variations in anesthesia level (115). In pediatric patients, implantation is usually performed under general anesthesia in a one or two step procedure (116). Clinical improvement in acquired dystonia is often delayed and becomes apparent only after weeks or even months. For instance, in a study on adult patients, the best therapeutic effect was observed not until 1.5 years after implantation (79). Therefore, optimal electrode placement is particularly challenging in pediatric patients with acquired dystonia. Figure 1 illustrates the placement of electrodes in the GPi.

A systematic approach to DBS programming can be useful to maximize functional benefit while minimizing adverse effects (117). According to published suggestions, after the initial monopolar review, therapeutic contacts should be regularly tested with gradually increasing stimulation intensity, with maximum amplitude or voltage set at least 0.2 mA or V below the threshold causing unwanted side effects. Lead impedances should be checked regularly to detect open or short circuits. Software tools, including 3D reconstructions and diffusion MRI-based tractography, is increasingly applied and can support optimal contact selection. However, the experience with their application in pediatric patients is limited so far (118).

In addition to structural imaging, neurophysiological methods can elucidate functional alterations within the cortical and subcortical motor networks associated with dystonia and DBS effects. Analyses of cortical oscillations using transcranial magnetic stimulation (TMS) and pallidal local field potential (LFP) recordings reveal altered activity patterns in isolated generalized dystonia, some of which correlate with symptom severity and provide valuable insights into the underlying pathophysiology of dystonia (119, 120). Novel DBS devices enable the recording of neuronal activity through the implanted leads during stimulation, allowing to correlate therapeutic effects with disease-specific oscillatory patterns, such as beta-band activity (13–30 Hz), which is suppressed by effective stimulation in Parkinson’s disease (121). In dystonia, pallidal beta burst activity has been linked to bradykinesia, suggesting that beta oscillations may serve as a marker of hypokinesia across movement disorders (122).

Long-term efficient stimulation settings seem to play a crucial role in therapeutic outcome in view of a substantial number of patients classified as “non-responders,” that is, DBS effects less than 20% assessed by the BFMDRS-M, or with declining effects over time (89, 123), potentially due to tolerance development or insufficient adaption of programming. These observations emphasize the importance of continuous monitoring and individualized optimization of stimulation parameters. Therefore, regular clinical follow-up with systematic stimulation adjustment, but also the early integration of rehabilitative measures seem to be essential for achieving long-term treatment success (124). Equally important is the psychosocial dimension of aftercare, including neuropsychological support and counseling for family members. Long-term follow-up studies have shown that adverse events are common and underline the need for structured follow-up management (124, 125), including early detection and treatment of stimulation-related side effects and hardware complications.

### 2.6 Age at implantation

Currently, DBS hardware is licensed from the age of 7 years, therefore implantations are typically performed in school-aged children (126). There is ongoing debate about whether surgical intervention during an early stage of development offers advantages in terms of neuroplasticity and functional outcomes. In a study investigating patients with genetic dystonia, younger age at surgery (<27 years) and shorter disease duration (<17 years) were associated with the best DBS outcome, with mean BFMDRS-M improvements of 25% after 1 year compared to older patients with longer disease duration (127). Whereas in a pediatric cohort of patients with genetic and acquired dystonia DBS response declines with the proportion of life lived with dystonia, emphasizing an intervention within 5 years of onset to maximize benefits and minimize the risk for orthopedic deformations (87). However, implantations during early childhood may increase the risk of adverse events, such as infections, hardware problems, or lead dislocation due to growth (116, 125, 126, 128). The youngest patients so far reported to have undergone DBS were 3.5 years old (20, 61, 129).

During the decision process several ethical considerations need to be weighed up including the lack of prognostic markers for patients with acquired dystonia and the risks of over- or undertreatment of pediatric patients: The decision for or against an elective surgical intervention in children may be influenced by various factors, including organizational or caregiving considerations. Undertreatment may occur if patients are unable to adequately express their own preferences or symptoms due to impaired communication abilities or cognitive impairment. In such cases, it is crucial to ensure decision-making processes in a way that best represents the child’s individual interests and quality of life (126).

### 2.7 Adverse events

Adverse events associated with pediatric DBS, including surgery-, hardware-, and stimulation-related complications, are common and are summarized in Table 3. Surgery-related adverse events (AEs) in pediatric DBS are primarily linked to electrode and IPG implantation or anesthesia and typically occur intraoperatively or within the first postoperative weeks. According to the GEPESTIM registry, among 72 children undergoing DBS up to the age of 18 years, 20 reversible and 2 irreversible AEs (wound infections requiring surgery) were reported within the first 4 weeks postoperatively (125). Severe adverse events in pediatric cohorts include asymptomatic hemorrhage (0.8–1.4%) and epileptic seizures (1.4%) during or after lead implantation (130–132).

**Table 3:** Adverse events (AE) associated with deep brain stimulation.

Surgery-related AEs	Hardware-related AEs	Stimulation-related AEs
Infection	Hardware infection	Dysarthria
Intra- or extracranial hemorrhage	IPG malfunction	Dysphagia
Seroma/CSF leak	IPG migration	Dyskinesia
Dehiscence/erosion of wound	Electrode or extension lead displacement	Increased muscle tone
Pain	High impedances due to electrode or extensions lead fractures or short circuit	Muscle contractions
	Extension lead malposition/migration	Visual disturbances
	Shortening of the extension lead due to growth	Paraesthesia
	Accidental switching-off of the device	Bradykinesia
	Battery expiry	Gait or balance disturbances
	Re-charging problems	Pain
		Discomfort
		Nausea

CSF, cerebrospinal fluid; IPG, implanted pulse generator.

According to a larger cohort of 129 pediatric patients, surgical-site infections within the first 6 months after implantation occurred in approximately 10% of cases, often requiring complete removal of the DBS hardware despite antibiotic treatment (130). In a cohort of 72 pediatric patients, 2 perioperative infections occurred within 4 weeks postoperatively and wound related AEs between 1 and 6 months postoperatively were reported in 12.5%, including 3 irreversible events (125). Infection rates are higher in children than in adults, especially in patients with acquired dystonia. Most hardware-related infections occur in the early postoperative period, with reported incidences ranging from 4.7 to 57% depending on age and type of device (116, 125, 130). *Staphylococcus aureus* is the most frequently isolated pathogen (133–135).

Due to the fact that growth is still ongoing, lead dislocations can occur and hardware revisions are more likely. Regular clinical follow-ups, precise lead placement with spare length of the extension lead, sufficient pain management, including regular analgesics during the first 2–3 days, and standardized perioperative antibiotic regimens (e.g., broad-spectrum cephalosporin for 5–7 days) are essential to minimize these risks.

Hardware-related problems in pediatric DBS primarily involve the implantable pulse generator (IPG) and the leads. Accidental IPG switch-off occurs in up to 18.7% of cases, usually due to incorrect handling or insufficient recharging (130). Children with DCP appear particularly prone to lead fracture, dislocation, or tension during growth, which has been hypothesized to relate to increased mechanical stress on implanted components by the hyperkinetic and hypertonic movements (125, 130).

Stimulation-related AEs are common but usually reversible by adjusting DBS parameters. Patients may experience dysarthria, increased tone, dyskinesia, paresthesia, discomfort, or visual disturbances, which typically resolve after reducing or switching off the stimulation. Lack of therapeutic effect, or “non-response,” can be associated with suboptimal lead placement, dystonia etiology, or progression of the underlying disease, emphasizing the importance of thorough patient selection, including genetic testing. Regular visits for clinical evaluation, effective adjustment of stimulation parameters using imaging- or tractography-based software and monitoring of lead impedances is essential to maximize efficacy, and minimize unwanted side effects. Repeated training of the families to educate about device management are crucial, particularly in pediatric populations in order to prevent acute deterioration from accidental IPG switch-off or battery depletion.

### 2.8 Variability of effects

The therapeutic response to DBS in patients with acquired dystonia shows considerable interindividual variability. The main causes for the variability in outcome are the heterogeneity of the cohorts investigated in terms of clinical phenotype, extent of brain damage, etiology of dystonia, choice of DBS target and stimulation parameters, time of follow-up, as well as the variability in outcome measures, which mainly focus on dystonia severity and impairment, rather than investigating patient-centered treatment goals (11, 127). In addition, with the increasing application of next-generation-sequencing (NGS) techniques pathogenic or likely pathogenic variants are sometimes identified, which can coexist with additional intrinsic and environmental antenatal and perinatal risk factors, supporting multifactorial causal pathways. This genetic and non-genetic heterogeneity further contributes to the variation in therapeutic response. Furthermore, NGS can uncover cases in which the diagnosis is not true CP but rather a CP mimic, such as dopa-responsive dystonia or hereditary spastic paraplegia. In such cases, the expected response to DBS may be absent and alternative treatments may be indicated (136, 137).

Patients with widespread or multifocal lesions, as commonly found in DCP, generally respond less well to DBS than patients with normal MRIs (81). Non-responders frequently exhibit extensive bilateral lesions, e.g., in the thalamus and cortical regions. Accordingly, they often present a heterogenous phenotype with dystonia and other neurological symptoms like spasticity or ataxia (138). Therefore, it is essential that dystonia is clearly identified as the predominant symptom and reliably distinguished from other neurological symptoms.

Patients with genetic or idiopathic dystonia present a distinctively lower mean preoperative BFMDRS-M (70), compared to patients with acquired dystonia, who are usually more severely affected (16, 128). This higher baseline disease burden might influence postoperative outcomes, too.

Other unfavorable predictors may include pronounced cognitive impairments and behavioral disturbances (77, 79, 139, 140). Comorbidities, including neuropsychiatric disorders, and particularly severe dystonia might negatively impact the patient’s psychological and functional adaption after surgery, thereby impairing postoperative adjustment, rehabilitation capacity, and the perception of DBS benefits (124, 141). Several other exclusion criteria have been discussed in previous publications, such as progressive neurodegenerative diseases, uncontrolled epilepsy, extensive cortical lesions, severe psychiatric comorbidities and acute infections (16, 97, 124, 142, 143).

Overall, according to previous reports, typical responder characteristics are reported to be phasic instead of tonic dystonia, focal or segmental distribution of dystonia instead of generalized, preserved cognitive function, minimal or no accompanying spasticity, the absence of orthopedic deformities, the lack of or at least only minor structural lesions typically localized to basal ganglia, and a shorter disease duration (55, 82, 87, 127, 142, 144–146).

## 3 Conclusion and future perspectives

The interpretation of the available evidence on DBS in acquired dystonia is complicated by several methodological limitations. The pronounced heterogeneity of the study populations, with different etiologies, variable lesion patterns, and a broad spectrum of accompanying motor and non-motor symptoms, make comparability difficult. Many studies include only small sample sizes, limiting statistical power and not allowing a reliable subgroup analysis.

In the future, larger multicenter studies on homogeneous cohorts are needed to pave the ground for more robust evidence. They should employ sensitive patient-centered outcome measures that capture functional, psychosocial, and participatory aspects in addition to motor symptoms and ideally include controlled comparisons with natural history cohorts to evaluate the long-term effects of DBS on motor and non-motor domains. In line with the International Classification of Functioning, Disability and Health (ICF), DBS outcomes should be evaluated from a multidimensional perspective, encompassing body functions and structures (e.g., dystonia severity, speech and swallowing, sleep quality, cognitive function), activities (e.g., self-care, fine motor tasks), participation (e.g., school, work, social engagement), as well as environmental and personal factors. This perspective highlights clinically meaningful improvements that might be missed by focusing on dystonia severity alone. The integration of objective measurement methods such as wearables or machine learning-based video analysis systems for classifying movement patterns and reflecting everyday fluctuations, might further improve the validity and reliability of outcome assessment in patients with hyperkinetic and hypertonic movement disorders (97, 147, 148).

Further studies integrating lesion mapping, connectomics and clinical phenotypes are needed to better understand the underlying pathophysiology and to identify potential targets for DBS.

Network-based planning of electrode placement is becoming increasingly important, and particularly relevant for patients with structural brain damage (26). Tractography and functional network analyses allow individual neuroanatomical characteristics to be taken into account and stimulation to be tailored to relevant motor circuits (109, 149). Innovative technologies such as AI-supported image analyses also offer the possibility of systematically analyzing complex imaging data and automatically identifying predictive patterns or optimal stimulation parameters (150). These could complement manual programming in the future, thus enabling more individualized and efficient therapy adaptation (151–154). Most of the required software tools are commercially available, however the application is still limited in pediatric patients so far (118).

The identification of disease-specific electrophysiological markers through TMS or LFP recordings provides promising opportunities to optimize individualized target selection and stimulation settings in dystonia. Adaptive (closed-loop) DBS systems integrating such neurophysiological feedback may enhance therapeutic efficacy, reduce side effects, and provide prognostic information regarding the expected outcome of DBS (105, 119, 120, 146, 155–159).

So far, DBS has mainly been applied in patients with DCP who commonly have a very low functional level (GMFCS IV/V) (88). With increasing knowledge of the underlying pathophysiology, improved understanding of target selection and stimulation effects, and better predictability of treatment outcome as well as a distinctively low risk of perioperative AEs, DBS could potentially be extended to children with moderate symptoms and might even become a first-line treatment in selected cases in the future (141).

Overall, DBS can be efficient for some patients with acquired dystonia, but the outcome is very heterogeneous and markedly lower than in genetic forms of dystonia. It is important to note, that DBS does not improve other motor impairments such as weakness, spasticity, or ataxia, which may continue to restrict functional outcomes even if dystonia did improve (141). Due to the lack of solid outcome predictors, consultation of patients and families remains challenging and the indication for DBS in acquired dystonia remains a very individual decision. Therefore, clinical parameters, electrophysiological markers and imaging data should be systematically incorporated into decision pathways. Predictive scores or AI-supported decision models could contribute to improved identification of suitable candidates and allow personalized DBS treatment (149).
